# Heavy Foils and Heavy Mallets

**Published:** 1870-11

**Authors:** G. H. Cushing


### ARTICLE III.

### *Heavy Foils and Heavy Mallets.*

By G. H. Cushing, D. D. S.

**Read before the Illinois State Dental Society.**

The title of the paper assigned to me presents two entirely distinct subjects, which will of course be considered separately, and in the order in which they are named.

The subject of "heavy foils," presents for our consideration the most remarkable and complete revolution in ideas and practice which has ever occurred in the dental profession; one which, had it been predicted three months before it occurred, would have been scouted as utterly visionary and impossible by many, if not all, who were soon thereafter its first and most earnest advocates.

The tendency for many years past has been towards lower numbers that is,to lighter foils; till No. 2,or that which contained but two grains to the sheet, was considered by many as the only foil which should be used by those desiring to secure the very best results. Now, when we come to the proposition, at once and with no intermediate steps, that No. 60, or foil weighing sixty grains to the sheet, is the best under most circumstances, and in fact the only style of foil which can produce first class results, we can realize something of the magnitude of the revolution we are about to discuss.

But more astonishing things have been known in the past, and we need spend no time in wondering at this extraordinary step in advance, if such it should prove to be, but at once proceed to the endeavor to understand the philosophy of this claim in favor of heavy foils, and to try and show what practical experience has demonstrated regarding their use. It will probably appear, upon a careful consideration of the subject, that there are good philosophical grounds upon which to base the claims made for these foils, and the absurdity of the proposition to substitute Nos. 30, 60 and upwards, for Nos. 2 to 6, will under this process almost disappear, while the fact which practical demonstration has established will confirm the theories.

In treating this subject it will be upon the assumption, of course, that the method of filling is by the use of adhesive foil exclusively. The requisites of a foil for this style of operation are, that it shall be as soft and pliant as possible, consistent with its great adhesiveness, and at the same time that it shall be tough and tenacious, not liable to disintegration afterwards. Perfectly pure gold, properly treated and rendered as thin as possible by rolling, probably possesses these qualities in the highest degree, while lamination, or beating out by the ordinary process, seems to deprive it in a measure, of these properties, particularly after the lower numbers are reached. The best article which has, perhaps, been published touching the philosophy of this portion of the subject, is one from the pen of Professor Eames, of the Missouri Dental College, which appeared in the January number of the "Missouri Dental Journal," and to which I refer you for a more full statement regarding this feature. It has doubtless been observed by many practitioners, that the lower numbers of foil, of all makers, did not possess the requisite of a first class adhesive foil, to the same extent as the higher numbers—I refer to Nos. as high as 8 to 10.

In conversation with Dr. W. H. Morgan, of Nashville, some two months ago, he stated that he had observed this peculiarity for a long time, and had been gradually using higher numbers, in special cases up to Nos. 8 and 10, but at the time of this conversation, he had not become a heavy foil advocate, as that term is now understood, so that it would appear that the minds of some of the profession were turned in this direction long ago.

Who was the first to reason the matter out to its ultimate conclusions, I am not aware. So far as we are informed, Dr. W. H. Atkinson, of New York, first called the attention of the profession generally to the matter in this country, and Dr. H. J. McKellops, of St. Louis, was the first to introduce the practice in the West.

I am informed by Dr. Horace Enos, of Chicago, that Dr. Evans, of Paris, has been using foil as heavy as No. 120, since, perhaps, six years ago, and we may yet learn that others of the profession in the old country, have also antedated us in the use of heavy foils. But it was immaterial who was first, and as we have no account, so far as I am aware, of the reasoning on the subject, which brought the early advocates to arrive at their *highest* conclusions, we may endeavor to reason for ourselves upon this point. It is clear that if Nos. 8 and 10 were found, by experience, to be better than the lower numbers, in respect to adhesiveness, then higher numbers still, might reasonably be supposed to possess, in a greater degree, this essential quality; and if it was found that Nos. 8 and 10 were applicable in cases where before it was deemed that they could not be successfully used, it was then equally reasonable to suppose that still thicker gold might be employed in such cases, also, advantageously.

These points could only be demonstrated by experiment, and so those first giving attention to this subject may be supposed, under the impulse of the above reasoning, to have tried experimentally heavier and still heavier foils, till the utmost limits were reached, and to-day we have some of the best men in the profession, advocating the use of foils of numbers raging from 60 to 120, and in some especial cases as high as 240.

Now, all that can be said in favor of this radical change in practice, would fail probably to convince the skeptical minds of the profession immediately. It is hardly to be expected that all the teachings of the advanced men of our profession, for these many years past, are at once to be set aside as all incorrect, and a new theory, so widely at variance with the old, to be accepted upon the mere affirmation of its advocates. We are, most of us like unbelieving Thomas, and can only be convinced by tangible proof and occular demonstration.

Still I will endeavor to state briefly, the grounds which I conceive to be philosophical, in support of the claims which are advanced for heavy foils, and also, what experience seems to have established regarding their practical use.

To reconcile the claims of this great revolution in practice with the principles of philosophy, will be to make clear to our minds, the proposition that foil of the thickness of No. 60, is better than No. 2, for the purpose filling teeth, that it is equally manageable, is applicable in all cases where No. 2 was formerly considered to be almost the only thing that could be used, that it is more adhesive, welding more perfectly, with less force, and making a more solid filling. I think this can be established. In order to do this, we must go back to the examination of how No. 2, or other low numbers of foil are manipulated, and in doing this, I think we can easily explain away a fallacy which has prevailed regarading the use of low numbers of foil generally.

The lower numbers of adhesive foil are used by either rolling it into pellets of various sizes, into ribbons or ropes of varying diameters and lengths, or folded upon itself with more or less irregularity into strips or blocks. I think it may be safely stated that the foil as it is generally prepared for use, presents a mass folded upon itself more or less irregularly, varying from ten to twenty thicknesses of the original foil, according to to the sized pellet, strip or block. Now, when we use this are we not practically using Nos. 20, 40 or 60? In fact, we are using a heavier foil than the number of folds represents, for it is so folded upon itself in such irregular shape that presents an endless succession of surfaces and angles, braced in every conceivable direction, one against the other, rendering the mass more resisting than a solid sheet would be, which represented the number of folds we have in our pellet.

Herein, I think, lies the fallacy which has prevented many from seeing anything reasonable in the claims made for the heavy foils, viz: in ignoring the fact that we have practically been using heavy foils, though composed of a succession of layers of lighter numbers; and I think it must be readily seen that if we can pack twenty layers of No. 2 or 3, satisfactorily, we can equally well pack a single layer of No. 40 or 60, in the same place, and it certainly is sound philosophy to say that it can be packed more easily and perfectly, for the reasons mentioned before, viz: that in folding the lighter numbers upon themselves, no matter how regularly, we have a largely increased resistance to overcome, and largely multiplied surfaces to weld together.

Now, as it is well known that the less gold is hammered or beaten, the more it retains of the properties of adhesiveness, softness, tenacity, and pliancy, I claim that philosophy clearly establishes that the thicker the gold, (within certain limits,) which can be used, the more perfect will be the welding, and the more solid will be the filling. Thus much for the philosophy sustaining these claims.

Now, to consider briefly, what experience teaches concerning the use of heavy foils:

1st. That as high as No. 60, they are applicable in all classes of cases, where the mallet can be used, even in the most frail teeth, where the inexperienced in the use of these foils would think they surely could not be used; but experience seems to prove that they are peculiarly adapted for use against frail walls, accommodating themselves to their irregularities with great facility, and requiring but slight force to place them properly, and in fact giving immediate support to the frailest wall, and thereby rendering the operation less difficult and less dangerous.

2d. No. 60 fills retaining points better than any othei number, making more certain and solid anchorages than the lighter foils.

3d. They are much more adhesive, and when properly packed must make a stronger as well as a more solid filling, while owing to the peculiar softness, as well as great adhesiveness of the numbers as high as 60 and upwards, they require really less force to condense them throroughly, than the numbers from two to ten.

4th. In binding they are invaluable; that is, where the cavity is approximal and the crown compounded, and the approximal portion can be but poorly anchored, as is sometimes necessarily the case, while a good anchorage can be secured in the crown—in such a case, by securing a strip of No. 60 or 120, in the crown, and carrying it over to the approximal portion, the filling can certainly be made more perfectly secure, than with light foils.

5th. In building out and restoring the contour, they make vastly stronger operations than anything of the kind done with the lower numbers could possibly make.

6th. They spread laterally, in condensing, to a much greater degree than the light foils; and, finally, to sum up in a word their merits, they produce when properly packed, the most perfect operations which have yet been attained.

Now, it may be said, and doubtless has been by many

"We must wait and see if these operations made with heavy foils fulfill the promise of their advocates, before we accept the new doctrine."

This is a very proper and natural caution, which we should all exercise, regarding new methods and practices, but I may remark, that it is very curious to observe how quickly these doubts disappear from the minds of the most skeptical when they have once given the matter a fair experimental trial.

I have three cases in my mind in which gentlemen experimented with the heavy foils, simply to prove their worthlessness. The foils were condemned in their minds beforehand, as utterly impracticable, and only tried by them at all, in order that they might be able to say, they had tried them, and the foils had proved just what they knew they would—worthless. All three of these gentlemen are now using the heaviest foils exclusively. The first experiments opened their eyes to the fact that their prejudices were unjust, and further experience only more and more fully establishes the superiority of the heavy over the light foils.

From my own experience, and that of others of my acquaintance, it would seem that all such doubts vanish before the "inexorable logic" of practical experiment. We are convinced in spite of ourselves, and the perfection of the operation of these foils becomes so established as we use them more, that there is not left any ground for even the most careful to say, "we must wait for time to prove the value or otherwise of this new method." I say this seems to be the case, and yet, perhaps, it is now to early too say that time will not prove that our present convictions are erroneous. My own belief is, that time will prove conclusively the truth of the seeming advantages of the new method.

With reference to the method of manipulation, it is difficult to write satisfactorily; one observatien of an operation would teach more than twenty pages of written instructions, and I will only briefly state some of what I consider the chief points to be closely observed. The foil should not be handled with uncovered fingers; it may be cut into pieces varying from one-fourth to three-fourths of an inch square, or into strips of proper width and length to suit the case. Where easy access is to be had to the cavity, the strips may be used to excellent advantage, condensing each layer across the whole surface, and folding the strip upon itself, thus saving time. The retaining points should be carefully filled, and the foil packed carefully from one retaining point to the other, securing and preserving as nearly uniform and plane a surface as possible, and the foil should be carefully condensed at and over the margins of the cavity, layer by layer. Care should be observed not to allow the foil to become very much crumpled or folded very irregularly upon itself. Rather small points should be used in packing it, as with them less force of malleting is required than with the larger points, while in finishing the operation of packing, great care should be observed to condense the entire surface thoroughly.

It must not be supposed that operations are greatly facilitated by using the heavy foils. My own experience thus far, is, that it requires longer to perform thorough operations with the heavy than with the light foils; but that should not weigh against them when the greater superiority of the operation is considered.

I have thus far spoken chiefly with reference to No. 60 and higher, which I consider as most desirable in a majority of cases, though Nos. 20 and 30 may be used to advantage in large fillings, for the principal part of the operation, as those numbers, when the cavity is favorable, can be packed more rapidly than the higher, while the heavier numbers should be used for the margin and finishing. I would also recommend these numbers to beginners, for their experiments, from which they will soon feel their way to the higher grades.

Before concluding what I have to say upon the subject of heavy foils, I would remark that since writing this paper, I

have been using foil of Nos. 30, 60, 120 and 240, which were not hammered at all, but produced by rolling, and they are certainly softer and more manageable than any I have used made by the ordinary practice of beating, and I think more adhesive. They seem to me, forcibly to corroborate the statement made in the first part of this paper, that "perfectly pure gold, properly treated and made thin by rolling, possesses the essential properties of an adhesive foil in the highest degree." This foil also possesses the advantage of presenting a dull surface, an advantage which those who have used the heavier bright foils will not fail to appreciate. R. S. Williams, of New York, is the manufacturer of the foils I now speak of.

In conclusion, I would earnestly commend the heavy foils to all who desire to secure the highest results, feeling confident that if these foils are fairly and properly tried, they will commend themselves far beyond anything that I could say in their favor.

### HEAVY MALLETS.

In speaking of the subject of heavy mallets I beg to say that when I proposed to write upon this, I hoped to be able to present some approximate explanation of the philosophy of the matter. But I shall make no such presumptous attempt.

Some articles have been written upon this subject, and from one by Prof. Watt, it would seem as though the matter might be easily explained. My own researches and experiments do not lead me to that conclusion, and I shall only very briefly refer to the philosophy of the subject, and state the results of the practical observations, and of some few experiments instituted in the effort to elucidate the subject.

The whole doctrine of "impact" is involved in this discussion, and it is one which it would seem is but little understood, even by scientific men. In my endeavors to get light upon this investigation, I wrote to the editors of the "Scientific American," who replied to me that they knew of no work treating of "impact" in other than a "mathematical way." They say "the subject is only amenable to mathematical investigation, and hence it is difficult to give a clear exposition in ordinary discussion."

But we can speak of the facts which observations and experience have established. What we require in a mallet is that it shall impact the gold properly, with the least possible injury to the tooth, and the parts surrounding it, and the least discomfort to the patient.

The theory of Prof. Watt, and probably of many others who have never used the heavy mallet is, that a light, elastic mallet is better than a heavy, non-elastic one, for the reason that the momentum of a light, elastic mallet, equaling the momontum of a heavy, leaden one, being secured by greater velocity, imparts its force so quickly that there is no time for its distribution to any extent beyond the tooth itself. Now, if the force were not diffused beyond the tooth at all, or were we operating upon mere inanimate matter, this theory would be all very well. But we are operating upon living, sensitive tissue, with all the physiological conditions qualifying it, and with the light mallet the force *is* diffused beyond the tooth, and that only enough to occasion sometimes great soreness of the periosteum, and to render the operation consequently very painful. Now the heavy leaden mallet, moving at much less velocity for the same momentum, has its force diffused very much more, so that in a certain sense, the jaw and the head divide the force of the blow with the tooth and its immediate surroundings.

Experience demonstrates that as a rule the heavy leaden mallet is by far the least disagreeable one to the patient, while impacting the gold equally as well as the light elastic mallet, and my theory is, that it is for this very reason, because the force in that case is more diffused than when the light elastic mallet is used ; and I think this is clearly reasonable, in view of what has been stated above.

But it does not appear why there should be so very great a difference. There seems to be something in the character of the blow dependent upon the material used, which has a modifying influence.

Mallets of almost every description have been used. Lead, steel, copper, wood, ivory, hard rubber, and rubber or wood faced mallets, with springs to break the force of the blow. You will find advocates for each and all these various kinds, but I feel very confident that when the matter has been fairly tested, the conclusion will be universally reached that an uncovered leaden mallet, weighing from six to eight ounces, will be preferred by nine-tenths of those upon whom they are used, and that the gold is equally as well and satisfactorily condensed.

I have personally tried faithfully the light wooden, the light leaden, the light hard rubber, the steel and the heavy leaden mallet; trying them all in the same mouth, and comparing them daily for several months, and have come to the conclusion stated above, that the heavy leaden mallet is universally preferred, except in very rare cases.

While attending the Mississippi Valley Dental Association held early in March of this year, Prof. Watt detailed experiments which he had been making, to demonstrate the action of different materials, and of different weights of the same materials as mallets, that is as to their relative "*vis viva*," or the amount of work which they would relatively accomplish in the way of impact before coming to a state of rest. The experiments which I afterwards instituted myself were suggested by those of Prof. Watt, and as I thought his apparatus not so constructed as to be uniformly accurate in its operation, I had mine made with a view to the greatest possible accuracy; and the results of my experiments satisfied me that the apparatus was all that could be desired for the purpose. It consisted of an upright board 18 inches high by 3 feet wide, set in a base of wood 8 inches wide, thus making a platform at the base of the board, in front, 3 inches wide. From in front of this board, at its central point, was suspended by means of an arbor running accurately in close fitting journals, a pendulum of steel wire, which could be lengthened or shortened at pleasure, and to which mallets could be attached, of any size or material.

Upon the platform at the base of the board, running in grooves of brass, its base resting on the platform of wood, was a brass box—weighted inside with lead—about 5 inches long by 2 inches high and 1½ inch wide, weighing altogether about three or four pounds; and upon the top of this brass box a steel bar was accurately fitted, to move lengthwise upon the box in nicely adjusted bearings; a set screw with a spring adjusted the tension of the bar at will. Opposite the bar and also the brass box, were fixed graduating scales of 24ths of an inch. These being set at zero, and so that the end of the bar should just touch the end of the mallet when it was hanging at rest, the mallet was drawn to a certain point and dropped, striking the end of the bar at a point of greatest momentum. The results of the blow were then evident of course; upon reading upon the graduating scales the distance passed over by both the bar and brass box, and thus was seen the relative effect of the blow upon the bar and the whole mass.

In this way, by comparing the results of blows produced by different mallets, was seen the difference in the diffusion of force due to the difference in the character of the material composing the mallet, as well as the difference of "*vis viva*."

The experiments were limited for want of time, and not of sufficiently varied a character to demonstrate all that we seek to know, but they were somewhat astonishing in some respects, and I think contrary to the generally accepted theories concerning impact. I will briefly detail the experiments, which were conclusive as far as they went. They were each repeated eight times or more with most *remarkably accurate* coincidence of results. But one blow of the mallet was struck in each experiment.

The spaces on the graduated scales will be designated as degrees.

Lead mallet. . . . . . . . . { drove the steel bar 4 degrees; whole mass .5 degrees.

| | |
|---|---|
| Lignum Vitæ, same weght. | steel bar 5 degrees; whole mass .1 degree. |
| Lignum Vitæ, with lead ends, same weight. . . . | steel bar 4 .5 degrees; whole mass .1 degree. |
| Soft iron, same weight. . . | steel bar 5 degrees; whole mass .4 degrees. |
| Soft pure rubber, same wt. | steel bar 4 .5 degrees; whole mass .4 degrees. |

The diffusion of force is apparent upon comparison of the distances passed over by the whole mass, and the steel bar moving upon the mass, the greater diffusion of course being where the whole mass was moved the greater distance relatively to the distance passed over by the bar in the same experiment. Thus, it will be observed, that in the first four experiments the lead seemed to exhibit the greatest diffusion, and the lignum vitæ the least, while the soft iron would seem to have the greatest amount of "*vis viva.*" In these four experiments then, it would seem to be fair to presume that the difference in the diffusion of force here evinced, was due to the difference in elasticity of material used as mallets; the more elastic showing less diffusion; but when we examine the record of the experiment with the soft rubber, we find that the diffusion of force in this experiment seems to be equal, if not greater, than in the case of the lead mallet, and its "*vis viva*" to bear the same relation to the lead that its diffusion does. This certainly seems strange, and I do not propose to account for these phenomena at present. I only give these experiments as being curious and interesting, and showing the importance of experimental research in such investigations, and also in the hope that what has been said upon the subject may stimulate others to investigate it. The length of this paper, so much beyond what I had intended it to be, I should deem required some apology, if I did not feel that although for practical purposes this physiological demonstration is not needed, yet it ill becomes a body claiming to be scientific, to rest short of a thorough philosophical and scientific investigation of all processes it may have occasion to use. In conclusion, I would state that the experiment with pure rubber surprised me greatly, but naturally suggested the inquiry in my mind, "if the action of the rubber in driving this bar so nearly approximates in results to the more solid, both elastic and non-elastic materials, why may it not be used to pack the gold as other mallets are? I made the experiment with it, having, however, little confidence that it would operate successfully for the purpose, and was much astonished to find, that apparently it effected the purpose equally as well as the other mallets, and in the case in which I used it, with much less suffering or annoyance to the patient. I only tried it in two cases, and cannot of course say whether it would generally be preferred, but I think it not improbable that it may prove valuable in certain cases of very lengthy operations, where the teeth are extremely sensitive to malletting with any material.

I propose to carry out these experiments in the future and hope when they are completed, to be able to say something more worthy of the subject.

---

